# A mathematical model of calcium dynamics in HSY cells

**DOI:** 10.1371/journal.pcbi.1005275

**Published:** 2017-02-15

**Authors:** Jung Min Han, Akihiko Tanimura, Vivien Kirk, James Sneyd

**Affiliations:** 1 Department of Mathematics, The University of Auckland, Private Bag 92019, Auckland 1142, New Zealand; 2 Department of Pharmacology, School of Dentistry, Health Sciences University of Hokkaido, Ishikari-Tobetsu, Hokkaido 061-0293, Japan; University of California San Diego, UNITED STATES

## Abstract

Saliva is an essential part of activities such as speaking, masticating and swallowing. Enzymes in salivary fluid protect teeth and gums from infectious diseases, and also initiate the digestion process. Intracellular calcium (Ca^2+^) plays a critical role in saliva secretion and regulation. Experimental measurements of Ca^2+^ and inositol trisphosphate (IP_3_) concentrations in HSY cells, a human salivary duct cell line, show that when the cells are stimulated with adenosine triphosphate (ATP) or carbachol (CCh), they exhibit coupled oscillations with Ca^2+^ spike peaks preceding IP_3_ spike peaks. Based on these data, we construct a mathematical model of coupled Ca^2+^ and IP_3_ oscillations in HSY cells and perform model simulations of three different experimental settings to forecast Ca^2+^ responses. The model predicts that when Ca^2+^ influx from the extracellular space is removed, oscillations gradually slow down until they stop. The model simulation of applying a pulse of IP_3_ predicts that photolysis of caged IP_3_ causes a transient increase in the frequency of the Ca^2+^ oscillations. Lastly, when Ca^2+^-dependent activation of PLC is inhibited, we see an increase in the oscillation frequency and a decrease in the amplitude. These model predictions are confirmed by experimental data. We conclude that, although concentrations of Ca^2+^ and IP_3_ oscillate, Ca^2+^ oscillations in HSY cells are the result of modulation of the IP_3_ receptor by intracellular Ca^2+^, and that the period is modulated by the accompanying IP_3_ oscillations.

## Author summary

We construct a mathematical model of Ca^2+^ and IP_3_ oscillations in HSY cells, a salivary ductal cell line from human parotid. The model reproduces the experimental data that exhibit coupled oscillations of [Ca^2+^] and [IP_3_] with the peak of each Ca^2+^ spike being followed by the peak of an IP_3_ spike. Recently, it was conjectured that IP_3_ oscillations in HSY cells are not necessary for Ca^2+^ oscillations. We corroborate this statement with our model and show that Ca^2+^ oscillations can occur without oscillating [IP_3_]. Further to this, based on our model simulation, we hypothesise that IP_3_ oscillations in HSY cells may affect the frequency of Ca^2+^ oscillations. Indeed, experimental data verify that oscillating [IP_3_] lengthens the period of Ca^2+^ oscillations.

## Introduction

Saliva secretion and regulation are vital for a range of activities, but can be compromised in a number of ways. Radiation therapy for head and/or neck cancer often causes salivary gland hypo-function, which can lead to xerostomia, commonly known as dry mouth [1, 2]. Patients with Sjögren’s syndrome also show symptoms of salivary gland dysfunction [3]. As saliva is directly linked with oral health and maintenance, lack of saliva is highly likely to cause oral pain, dental cavities and infections. Thus, it is important to understand the mechanisms underlying saliva secretion and regulation, in order, ultimately, to attempt to reverse the damage caused by salivary gland diseases.

There are three main salivary glands: parotid, sublingual and submandibular. The parotid glands are the largest pair, and each gland is structured like a bunch of grapes, with a network of ducts and a cluster of acinar cells on the ends. Generally, studies of saliva formation have focused on the understanding of acinar cells, as ductal cells are not the primary source of saliva secretion. However, Baum et al. [4] presented a gene therapy procedure that targets ductal cells, and successfully showed that it alleviated hyposalivation in rats and miniature pigs that were pre-exposed to radiation. In 2012, a clinical trial of the gene therapy showed that 6 of the 11 treated individuals had an increased level of saliva secretion, and five participants also experienced moisture and lubrication in their mouths [5]. Their findings demonstrated the necessity of investigating the mechanisms and involvement of ductal cells in saliva secretion and regulation.

It is well established that changes in intracellular calcium concentration ([Ca^2+^]) are important in both intracellular and intercellular signalling [6–13]. Douglas and Rubin [14] were the first to show that intracellular calcium (Ca^2+^) plays an important role in the saliva secretion process. They discovered the absence of cytosolic Ca^2+^ inhibits saliva secretion. Numerous studies reported the close linkage between intracellular Ca^2+^ signals and ion channels in salivary glands, including Cl^−^ channels [15–17], K^+^ channels [18, 19], and ${\text{Cl}}^{-}/{\text{HCO}}_{3}^{-}$ exchangers [20, 21]. These results emphasise the importance of studying the correlation between the behaviours of intracellular [Ca^2+^] and the functions of cells involved in the secretion and regulation of saliva.

Several studies show that when HSY cells, a salivary ductal cell line from the parotid gland, are stimulated with external agonists such as adenosine triphosphate (ATP) and carbachol (CCh), they exhibit oscillations in [Ca^2+^] [22, 23]. However, their exact function is less well understood.

There are two major pathways that govern Ca^2+^ oscillations in HSY cells: release and re-uptake of Ca^2+^ from the endoplasmic reticulum (ER), and fluxes across the plasma membrane. The release of Ca^2+^ from the ER is initiated by inositol (1,4,5)-trisphosphate (IP_3_) binding to IP_3_ receptors (IPR) on the ER membrane, which opens the receptor (which is also a Ca^2+^ channel) allowing the flow of Ca^2+^ out of the ER. When [Ca^2+^] is high, Ca^2+^ is pumped back into the ER or removed from the cell across the plasma membrane.

We aim to use the studies in HSY cells to try to understand better the possible mechanisms that control Ca^2+^ oscillations in duct cells. Although parotid duct cells differ from HSY cells in many respects, much more is known about the Ca^2+^ dynamics of HSY cells, which makes them a better candidate for modeling work. In this paper, we present a mathematical model that reproduces Ca^2+^ oscillations in HSY cells. In order to verify the model, we compare model simulations with experimental data, and make and test predictions. Our aim is to understand the mechanisms underlying Ca^2+^ oscillations in HSY cells, and how IP_3_ dynamics affects the oscillations.

## Materials and methods

### Media

Hanks’ balanced salt solution with Hepes (HBSS-H) contained 137 mM NaCl, 5.4 mM KCl, 1.3 mM CaCl_2_, 0.41 mM MgSO_4_, 0.49 mM MgCl_2_, 0.34 mM Na_2_HPO_4_, 0.44 mM KH_2_PO_4_, 5.5 mM glucose, 20 mM Hepes-NaOH (pH 7.4). Intracellular-like medium (ICM) contained 125 mM KCl, 19 mM NaCl, 10 mM Hepes-KOH (pH 7.3), 1mM EGTA, and appropriate concentrations of CaCl_2_ (330 *μ*M CaCl_2_ for 50 nM free Ca^2+^).

### Cell culture

HSY-EA1 cells were cultured in Dulbecco’s Eagle’s medium nutrient mixture F-12 Ham (Sigma) supplemented with 10% newborn calf serum, 2 mM glutamine, and 100 U/ml penicillin and 100 *μ*g/ml streptomycin, as previously described. These cells were grown in fibronectin-coated experimental chambers consisting of plastic cylinders (7 mm in diameter) glued to round glass coverslips. For monitoring changes in intracellular [Ca^2+^], cells were incubated with 2 *μ*M Fluo-3 acetoxymethyl ester (Dojin Chemicals, Kumamoto, Japan) in HBSS-H HBSS-H containing 1% bovine serum albumin (BSA) for 30 min at room temperature.

### Measurement of fluorescence

Experiments were carried out on a TE2000 inverted fluorescence microscope (Nikon Tokyo, Japan) with dual-source illumination system, in which two different light sources, a 150 W xenon arc light source U7773 (Hamamatsu photonics, Shizuoka, Japan) and a 100 W mercury light source (Nikon) were equipped. An experimental chamber was placed on the microscope, and fluorescence images were captured using a imaging system consisting of a C9100-13 EM-CCD camera (Hamamatsu photonics) and were analysed with AQUACOSMOS 2.6 software (Hamamatsu photonics).

IP_3_-induced Ca^2+^ oscillations were examined with cell-permeable caged IP_3_, iso-Ins(1,4,5)P_3_/PM(caged), (Alexks Biochemicals, Grunberg, Germany). In this experiments, cells were incubated with cell-permeable caged IP_3_ (2–10 *μ*M) and Fluo-3 (2 *μ*M) in HBSS-H containing 1% BSA for 30 min at room temperature. During monitoring Ca^2+^ responses with 490 nm light from the Nikon mercury light source, cells were also illuminated with 400 nm light that was derived from U7773 for the photolysis of cell-permeable caged IP_3_. These illumination lights were reflected by a 500 nm long pass dichroic beamsplitter, and exposed the chamber through a Nikon Plan Fluor 40x (numerical aperture, 1.3) or 60x objective oil immersion lens (numerical aperture, 1.2). In this experiment, 400 nm light was continuously exposed to a small area including several cells, and the strength of the light was controlled with the grating monochromator in U7773 and neutral density filters, so that the Ca^2+^ oscillations was induced in a constant frequency. The emitted light of Fluo-3 can passed the dichroic beamsplitter and 535 nm emotion filter, and detected by the EM-CCD camera. ATP, CCh, and U73122 were directly added to the chamber during the measurements.

### The calcium model

Many mathematical models have been developed to explain intracellular Ca^2+^ dynamics of various cell types. The details of these models are different, as each aims to describe the precise dynamics in a certain cell, but they share a primary goal of explaining how Ca^2+^ dynamics is regulated. For recent reviews of computational models of Ca^2+^ oscillations, see Dupont et al. [24] and Schuster et al. [25]. There are two main hypotheses that are thought to be involved in the formation of Ca^2+^ oscillations: the biphasic regulation of the IPR by Ca^2+^, and oscillations in IP_3_ concentration ([IP_3_]) driven by cross-coupling between Ca^2+^ and IP_3_. In the former hypothesis, Ca^2+^ activation and inhibition of the IPR regulate the periodic opening of the receptors, giving rise to Ca^2+^ oscillations in the absence of oscillating [IP_3_] [26, 27]. Cytosolic Ca^2+^ can cause the release of Ca^2+^ from the ER through a Ca^2+^-induced Ca^2+^ release (CICR) mechanism, by binding onto the activating site of the IPR on a relatively fast timescale. Subsequently, Ca^2+^ can also bind onto the different sites of the IPR on a slower timescale to inhibit the receptors. Numerous studies employed this mechanism to describe Ca^2+^ oscillations and waves observed in different cell types, including Xenopus laevis oocytes [27, 28], hepatocytes [29], and airway smooth muscle cells [30]. The models that generate Ca^2+^ oscillations through Ca^2+^ feedback on the IPR are called Class I models. The latter hypothesis assumes that Ca^2+^ is involved in the formation and degradation of IP_3_, leading to coupled oscillations of [Ca^2+^] and [IP_3_] [31, 32]. As positive feedback, Ca^2+^ can activate the enzyme that produces IP_3_, phospholipase C (PLC), to increase [IP_3_], which in turn stimulates the IPR to release Ca^2+^ from the ER. However, Ca^2+^ can also be involved in the degradation of IP_3_, by activating IP_3_ 3-kinase (IP_3_K), the enzyme that degrades IP_3_ to IP_4_. This forms a negative feedback on the Ca^2+^ release through the IPR. Other negative feedback processes may involve Ca^2+^ inhibition of the IPR or suppressed production of IP_3_ by protein kinase C (PKC) [32–34]. The models that incorporate positive and negative Ca^2+^ feedback on IP_3_ as the main oscillatory mechanism are called Class II models. Despite the distinctive difference in the fundamental mechanisms underlying Ca^2+^ oscillations, it is very hard to experimentally distinguish oscillations generated by one mechanism from those induced by the other.

Recent development of IP_3_ biosensors has enabled the monitoring of changes in [IP_3_] during Ca^2+^ oscillations, which led to the discovery of concurrently oscillating [Ca^2+^] and [IP_3_] in some cells [22, 33, 35, 36]. Several studies thoroughly investigated the effects of cross-coupling between Ca^2+^ and IP_3_ on intracellular Ca^2+^ oscillations, both experimentally and theoretically. Dupont et al. [35] proposed that IP_3_ oscillations observed in hepatocytes, presumably induced by Ca^2+^-dependent IP_3_ metabolism, are not required for Ca^2+^ oscillations. Furthermore, Bartlett et al. [34] observed that photolysis of caged IP_3_ can elicit Ca^2+^ oscillations in hepatocytes, without any contribution from PLC. Politi et al. [32] constructed a model of Ca^2+^ dynamics with positive and negative Ca^2+^ feedback on IP_3_ regulation, and found that these feedback mechanisms extend the range of oscillation frequencies. Interestingly, Gaspers et al. [33] found that positive Ca^2+^ feedback on IP_3_ is essential for generating low-frequency Ca^2+^ oscillations. These results are readdressed in the Discussion in more detail, as they are closely related to the results of this paper.

As experimentally observed intracellular Ca^2+^ behaviours exhibit stochastic events, it is becoming increasingly important to include stochasticity in Ca^2+^ models. Indeed, a number of studies have used stochastic models to explain intracellular Ca^2+^ dynamics [28, 37–40]. For a recent review of stochastic Ca^2+^ models, see Rdiger [41]. Although stochastic models can be useful to study intrinsically random activities of the IPR, Cao et al. [30] showed that deterministic models can make equally valid predictions about intracellular Ca^2+^ dynamics as stochastic models can. Thus, we aim to study Ca^2+^ dynamics in HSY cells using a deterministic model.

**Fig 1:**
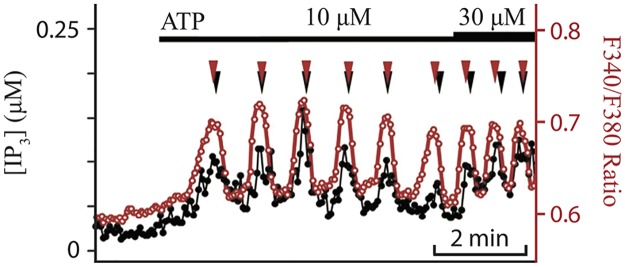
Estimated [IP_3_] (in black) and excitation ratio of fura-2 (in red) during ATP-induced Ca^2+^ oscillations in HSY cells. Traces are representative of 31 of 55 oscillating cells. The axis on the left is for [IP_3_] and the one on the right is for the ratio of fura-2. The black bar at the top shows the dosage of ATP used to stimulate the HSY cells. The triangles at the top indicate the peak of each spike. This figure was adapted from Tanimura et al. [22].

Tanimura and Turner observed that permeabilised HSY cells can exhibit repetitive Ca^2+^ release and re-uptake by intracellular stores, suggesting that Ca^2+^ oscillations are directly generated by Ca^2+^ feedback on IPR [42, 43]. Their work was later supported by Tojyo et al. [23], who presented evidence that Ca^2+^ oscillations in HSY cells, in the form of baseline spikes, can arise without activating IP_3_ formation. Subsequently, Tanimura et al. [22] monitored emission ratios of LIBRAvIIS and fura-2, biosensors of IP_3_ and Ca^2+^, respectively, and found that they exhibit coupled oscillations upon agonist stimulation with different concentrations of ATP (see Fig 1). This suggests that the model should contain Ca^2+^ feedback on IP_3_ production and/or degradation. Interestingly, the coupled oscillations showed a pattern where peaks of Ca^2+^ spikes preceded those of IP_3_ spikes. However, it was not known whether the IP_3_ oscillations were necessary in order for Ca^2+^ oscillations to exist, or whether they were simply a passive reflection of the Ca^2+^ oscillations. In this section, we construct a mathematical model of Ca^2+^ dynamics in HSY cells based on the experimental observations. The basic assumption of our model is that the oscillations arise via a Class I mechanism, in which the IP_3_ oscillations are simply a passive reflection of the Ca^2+^ oscillations. As we shall see, this assumption is upheld by experimental testing of model predictions.

#### Calcium dynamics

**Fig 2:**
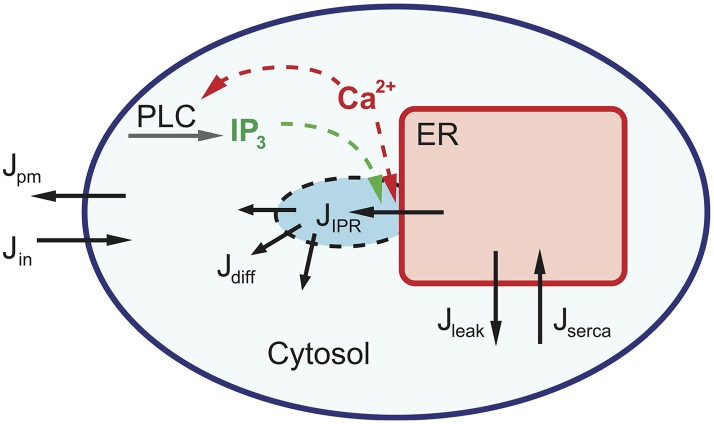
Schematic diagram of calcium fluxes in a cell. The directions of the fluxes are indicated with the arrows. When an IP_3_ receptor is opened, Ca^2+^ is first released from the ER to a nearby domain (*J*_IPR_), then diffuses to the rest of the cytosol (*J*_diff_). Eventually, Ca^2+^ is pumped back into the ER (*J*_SERCA_). There are fluxes across the plasma membrane (*J*_in_ and *J*_pm_), and a small leak from the ER to the cytosol as well (*J*_leak_).

We separate the cell domain into three compartments: inside the ER, a small region (microdomain) near a cluster of IPR, and the cytosol (see Fig 2); the Ca^2+^ concentration in each region is denoted by *C*_ER_, *C*_*b*_ and *C*, respectively. The total free Ca^2+^ concentration within a cell, *C*_*t*_, is given by

$$C_{t}=C+\frac{C_{b}}{\gamma_{1}}+\frac{C_{\text{ER}}}{\gamma_{2}},$$

where *γ*_1_ and *γ*_2_ are volume ratios between different compartments, as given in Table 1. Thus, *C*_ER_ = *γ*_2_(*C_t_* − *C* − *C_b_*/*γ*_1_). IPR interact with *C*_*b*_ and the [Ca^2+^] at the mouth of an open channel (denoted by *C*_*p*_); thus there is no direct interplay between the open probability of the receptors and the cytosolic Ca^2+^. The idea of the microdomain around the mouth of an IP_3_ receptor, or around a cluster of IPR, was first introduced by Swillens et al. [44], where they assumed that this domain, which is separate from the cytosol and the ER lumen, contains the receptor’s activating and inhibiting Ca^2+^-binding sites. Assuming that the receptor activity is modulated by the [Ca^2+^] in the microdomain, they were able to reproduce experimental observations where increments of IP_3_ induce repeated transient release of Ca^2+^. Dupont and Swillens further showed that the model could account for Ca^2+^ oscillations as well [45]. In addition, they discovered that one of the outcomes of the presence of a microdomain is an increase in the oscillation period.

**Table 1:** Parameter values of the Ca^2+^ fluxes.

Parameter	Value (Units)	Description
*γ*_1_	100	the volume ratio between cytosol and microdomain
*γ*_2_	10	the volume ratio between cytosol and ER
*k*_IPR_	0.038 (s^–1^)	IPR flux coefficient
*k*_diff_	10 (s^–1^)	Ca^2+^ diffusion rate coefficient
*k*_leak_	0.0032 (s^–1^)	ER leak flux coefficient
*V*_S_	10 (*μ*M s^–1^)	maximum SERCA flux
*K*_S_	0.24 (*μ*M)	half-maximal activating cytosolic [Ca^2+^] of SERCA
*J*_leakin_	0.0019 (*μ*M s^–1^)	plasma membrane leak influx
*V*_ROCC_	0.03 (s^–1^)	ROCC flux coefficient
*V*_SOCC_	0.3 (*μ*M s^–1^)	maximum SOCC flux
*K*_SOCC_	100 (*μ*M)	SOCC dissociation coefficient
*V*_pm_	0.033 (*μ*M s^–1^)	maximum plasma membrane efflux
*K*_pm_	0.45 (*μ*M)	half-maximal activating cytosolic [Ca^2+^] of PMCA

When Ca^2+^ is released through IPR, *C*_*b*_ increases to a high concentration essentially instantaneously. Ca^2+^ in the microdomain then diffuses to the cytosol, and eventually gets pumped back into the ER via sarco/endoplasmic reticulum Ca^2+^-ATPase (SERCA) pumps or removed from the cell by the plasma membrane Ca^2+^ ATP-ase (PMCA) pumps. Based on the schematic diagram of calcium dynamics in Fig 2, we write the following ODEs,

$$\frac{dC}{dt}=J_{\text{diff}}+J_{\text{leak}}-J_{\text{SERCA}}+J_{\text{in}}-J_{\text{pm}},$$ (1)

$$\frac{dC_{t}}{dt}=J_{\text{in}}-J_{\text{pm}},$$ (2)

$$\frac{dC_{b}}{dt}=\gamma_{1}\left(J_{\text{IPR}}-J_{\text{diff}}\right).$$ (3)

The functional form for each flux is specified in the next section.

#### Calcium fluxes

In our model, the flux through the IPR is given by

$$J_{\text{IPR}}=k_{\text{IPR}}O_{\text{IPR}}\left(C_{\text{ER}}-C_{b}\right),$$

where *O*_IPR_ is the open probability of the IPR. The flux from the microdomain to the cytosol is assumed to be a linear function of the concentration difference, and thus

$$J_{\text{diff}}=k_{\text{diff}}\left(C_{b}-C\right).$$

There is also a small leak flux across the ER membrane, modeled by

$$J_{\text{leak}}=k_{\text{leak}}\left(C_{\text{ER}}-C\right).$$

We express the re-uptake flux of Ca^2+^ via SERCA pumps as in [46],

$$J_{\text{SERCA}}=\frac{V_{S}C^{1.75}}{C^{1.75}+K_{S}^{1.75}}.$$

The total intracellular Ca^2+^ concentration is controlled by influx (*J*_in_) and efflux (*J*_pm_) across the cell membrane. *J*_in_ consists of a constant basal leak (*J*_leakin_) and fluxes through receptor-operated Ca^2+^ channels (ROCC) and store-operated Ca^2+^ channels (SOCC), denoted by *J*_ROCC_ and *J*_SOCC_, respectively. We model these fluxes as in [47],

$$\begin{array}{ccc} J_{\text{ROCC}} & = & V_{\text{ROCC}}\cdot P,\\ J_{\text{SOCC}} & = & V_{\text{SOCC}}\cdot K_{\text{SOCC}}^{4}/\left(K_{\text{SOCC}}^{4}+C_{\text{ER}}^{4}\right), \end{array}$$

where *P* is the intracellular [IP_3_]. Thus, the total Ca^2+^ influx across the plasma membrane is:

$$J_{\text{in}}=J_{\text{leakin}}+J_{\text{ROCC}}+J_{\text{SOCC}}.$$

*J*_pm_ is the Ca^2+^ efflux through the PMCA, and we follow [48] by modeling it as

$$J_{\text{pm}}=\frac{V_{\text{pm}}C^{2}}{C^{2}+K_{\text{pm}}^{2}}.$$

Parameters used in the Ca^2+^ fluxes are shown in Table 1, along with their descriptions.

#### Cell volume and calcium buffering

Rat parotid ductal cells show transient swelling during onset of Ca^2+^ oscillations, perhaps due to ion absorption [49]. However, it seems unlikely that Ca^2+^ oscillations in duct cells are dependent on an oscillating volume. In our model, we assume constant cell volume and aim to generate Ca^2+^ oscillations that are independent of changes in cell volume. Also, our model includes implicit calcium buffering as in Gin et al. [50], by assuming that buffers are fast, immobile and unsaturated.

### IP_3_ receptor model

**Fig 3:**
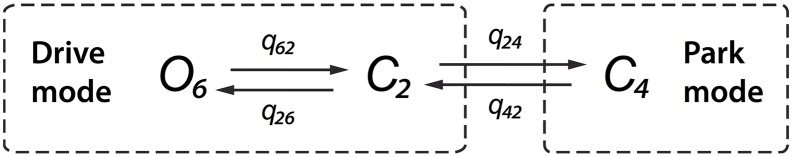
The structure of the IPR model. The model is comprised of two modes. The drive mode has one open state and one closed state, and has an open probability of *q*_26_/(*q*_26_ + *q*_62_). The park mode has one closed state with an open probability close to zero. *q*_24_ and *q*_42_ are the transition rates between the modes.

A crucial part of the model is our choice of the model for the IPR. We use the model of Cao et al. [30], which is an improved version of the Siekmann IPR model from [51]. The model consists of two modes: the drive mode and park mode; see Fig 3. The receptors are mostly open when they are in the drive mode, and closed when in the park mode. The drive mode has one open state (*O*_6_) and one closed state (*C*_2_), with transition rates *q*_26_ and *q*_62_ as shown in Table 2. Hence, the open probability of the drive mode is *q*_26_/(*q*_26_ + *q*_62_) (≈ 70%). The park mode has one closed state (*C*_4_), with an open probability close to zero. Cao et al. denoted the transition rates between the modes as *q*_24_ and *q*_42_, given by

$$\begin{array}{ccc} q_{24} & = & a_{24}+V_{24}\left(1-m_{24}^{\infty }h_{24}^{\infty }\right),\\ q_{42} & = & a_{42}+V_{42}^{}m_{42}^{}h_{42}^{}. \end{array}$$

If *D* is defined to be the proportion of the IPR that are in the drive mode,

$$D=\frac{q_{42}\left(q_{62}+q_{26}\right)}{q_{42}q_{62}+q_{42}q_{26}+q_{24}q_{62}},$$

then the IPR have an open probability as follows:

$$O_{\text{IPR}}=\frac{q_{26}}{q_{62}+q_{26}}D.$$

The opening kinetics of the receptors is governed by the following:

$$\begin{array}{ccc} \frac{dm_{42}}{dt} & = & \lambda_{m_{42}}\left(m_{42}^{\infty }-m_{42}^{}\right),\\ \frac{dh_{42}}{dt} & = & \lambda_{h_{42}}\left(h_{42}^{\infty }-h_{42}^{}\right), \end{array}$$

where $m_{42}^{\infty }$ and $h_{42}^{\infty }$ are the quasi-equilibrium values of *m*_42_ and *h*_42_, respectively, and the λ’s are the rates at which the quasi-equilibria are approached,

$$\begin{array}{ccc} m_{42}^{\infty } & = & \frac{C_{b}^{3}}{C_{b}^{3}+k_{42}^{3}}\\ h_{42}^{\infty } & = & \frac{k_{-42}^{3}}{C_{b}^{3}+k_{-42}^{3}},\\ \lambda_{h_{42}} & = & (1-D)L+DH. \end{array}$$

We assume that λ_*m*_42__, *L*, and *H* are relatively small, as shown in Table 2, corresponding to slow opening kinetics of the receptor; see the Discussion for more detail about the dynamics of the IPR model. The closing kinetics is controlled by *m*_24_ and *h*_24_, which are assumed to be at their quasi-equilibrium values,

$$\begin{array}{ccc} m_{24}^{\infty } & = & \frac{C_{p}^{3}}{C_{p}^{3}+k_{24}^{3}},\\ h_{24}^{\infty } & = & \frac{k_{-24}^{2}}{C_{p}^{2}+k_{-24}^{2}}, \end{array}$$

respectively. *C_p_* = *C*_*p*0_(*C*_ER_/680) denotes the [Ca^2+^] at the pore. The expressions for the *V*’s, *a*’s and *k*’s are

$$\begin{array}{cccc} V_{24} & =62+880/(P^{2}+4) & a_{24} & =1+5/(P^{2}+0.25)\\ k_{24} & =0.35 & k_{-24} & =80\\ V_{42} & =110P^{2}/\left(P^{2}+0.01\right) & a_{42} & =1.8P^{2}/\left(P^{2}+0.34\right)\\ k_{42} & =0.49+0.543P^{3}/\left(P^{3}+64\right) & k_{-42} & =0.41+25P^{3}/\left(P^{3}+274.6\right) \end{array}$$

For full details of the IPR model derivation, refer to Cao et al. [30].

**Table 2:** Parameter values for the IPR model.

Parameter	Value	Parameter	Value
*q*_26_	10500 s^–1^	*q*_62_	4010 s^–1^
λ_*m*_42__	1 s^–1^	*L*	0.1 s^–1^
*C*_*p*0_	700 *μ*M	*H*	0.1 s^–1^

### Ca^2+^ feedback mechanisms on IP_3_ dynamics and IPR

As mentioned before, Class I models assume that Ca^2+^ feedback on the opening and closing kinetics of IPR is the main driving force behind Ca^2+^ oscillations. Intracellular Ca^2+^ can increase or decrease the open probability of an IPR by binding to its different binding sites. In this case, time-dependent Ca^2+^ feedback on IPR is necessary to generate Ca^2+^ oscillations. Conversely, Class II models assume that Ca^2+^ oscillations are the result of Ca^2+^ feedback on the production and degradation of IP_3_. As a positive feedback, Ca^2+^ can activate phospholipase C (PLC), and increase the rate of IP_3_ production. Ca^2+^ can also have a negative feedback on IP_3_ by activating IP_3_ 3-kinase (IP_3_K) to remove IP_3_. The increases and decreases in [IP_3_] are coupled with the open probability of the IPR, leading to oscillations in [Ca^2+^]. Whereas a Class II model requires oscillating [IP_3_] in order to give rise to Ca^2+^ oscillations, a Class I model does not, and hence Ca^2+^ oscillations can occur even at a constant [IP_3_].

Tanimura and Turner [43] permeabilised the plasma membrane of HSY cells, and applied IP_3_ to monitor changes in the [Ca^2+^] in the IP_3_-sensitive store. The addition of IP_3_ caused subsequent release of Ca^2+^ from the store, followed by re-uptake of Ca^2+^ via SERCA pumps. This Ca^2+^ release and re-uptake were repeatedly observed in the absence of Ca^2+^ buffer (EGTA) but disappeared in the presence of EGTA. Their result suggests that Ca^2+^ oscillations in HSY cells are mainly generated by feedback effects of Ca^2+^ on IPR dynamics. It was also hypothesised in Tanimura et al. [22] that, although there are IP_3_ oscillations in HSY cells, they are not crucial for Ca^2+^ oscillations. These observations indicate that the oscillations can be reproduced in a Class I model. Additionally, we want to model IP_3_ dynamics with positive and/or negative feedback from Ca^2+^ to ensure there are coupled oscillations. The order of [Ca^2+^] and [IP_3_] peaks, in which [Ca^2+^] peaks are followed by [IP_3_] peaks (see Fig 1), suggests that Ca^2+^ has positive feedback on IP_3_. For this reason, we conjecture that in HSY cells, the production rate of IP_3_ is an increasing function of [Ca^2+^], and thus each peak in [IP_3_] is caused by the preceding peak in [Ca^2+^]. In order to construct a model as simple as possible, and yet still capture the essence of the experimental data, our model includes Ca^2+^ feedback on the production of IP_3_ only, with a constant rate of IP_3_ degradation.

The production of IP_3_ can be processed by many different forms of PLC [52]. There is little information about the PLCs expressed in HSY cells. However, Nezu et al. [53] proposed that HSY cells exhibit two distinct pathways for IP_3_ generation. They studied HSY cells that were mechanically stimulated by poking the cell surface with a glass micropipette. They also studied the neighboring cells that were not directly stimulated with the micropipette. This type of stimulation may induce both Ca^2+^ release from the ER and extracellular Ca^2+^ entry through channels on the plasma membrane [54]. The cells showed waves of Ca^2+^ and IP_3_ upon stimulation, which started from the site of stimulation and propagated to the neighboring cells. The Ca^2+^ wave was observed in the absence of extracellular Ca^2+^. When the cells were pretreated with suramin, a blocker that inhibits the receptors on the plasma membrane that are activated by external agonists to stimulate PLC, the responses in the neighboring cells were abolished. This observation suggest that the responses in the neighboring HSY cells were induced by the generation of intracellular Ca^2+^ and IP_3_ via a pathway that depends on external agonists. The responses in the cells with direct stimulation were not affected by suramin, which indicates that mechanical stimulation induces the generation of IP_3_ that is independent of external agonist. When the cells were pretreated with a PLC inhibitor, U73122, the mechanical-stimulation-induced Ca^2+^ and IP_3_ responses were suppressed. Additionally, HSY cells exhibited Ca^2+^ oscillations when stimulated with external agonist [22, 53]. These results suggested that there are at least two types of PLCs in HSY cells; one that is primarily independent of external agonist, and the other that is activated by the addition of agonist. When modeling PLC regulation in our model, we assume that HSY cells express PLC*δ*, a PLC that is regulated by Ca^2+^, and PLC*β*, another PLC that is activated by the addition of external agonist. Both the Ca^2+^ released from the ER and the Ca^2+^ that enters from the extracellular space contribute towards the activation of PLC*δ*. In our model, the concentration of applied agonist is denoted by *ν*. We model the IP_3_ production rate generated from PLC*β* as a saturating function, so that the rate of production increases as the agonist concentration increases, before reaching the maximum production rate [55]. The Ca^2+^ activation of PLC (PLC*δ*) is expressed with a Hill function [32, 50]. We model the production rate of IP_3_ as a sum of the rates generated from PLC*β* and PLC*δ*,

$$V_{\text{plc}}=\psi_{1}\frac{\nu }{K_{\nu }+\nu }+\psi_{2}\frac{C^{4}}{C^{4}+K_{\text{plc}}^{4}},$$ (4)

where *ψ*_1_ and *ψ*_2_ are the maximum production rate induced by PLC*β* and PLC*δ*, respectively. When *ψ*_2_ = 0 *μ*M s^–1^, IP_3_ production rate is solely from the agonist; when *ψ*_2_ ≠ 0 *μ*M s^–1^, an increase in [Ca^2+^] also increases IP_3_ production rate, leading to a positive feedback loop. We assume a constant degradation rate of IP_3_,

$$V_{\text{deg}}=r_{\deg}P,$$

where *r*_deg_ is the rate of decay. Thus, the ODE for [IP_3_] is,

$$\frac{dP}{dt}=V_{\text{plc}}-V_{\text{deg}},$$ (5)

with parameter values shown in Table 3.

**Table 3:** Parameter values for Eq (5).

Parameter	Value	Parameter	Value
*K*_*ν*_	45 *μ*M	*K*_plc_	0.8 *μ*M
*ψ*_1_	0.5 *μ*M s^–1^	*ψ*_2_	0.8 *μ*M s^–1^
*r*_deg_	1.4 s^–1^		

### The full model

Based on the above, we write the full HSY cell calcium model with six ODEs,

$$\begin{array}{c} \begin{array}{cc} \frac{dC}{dt} & =J_{\text{diff}}+J_{\text{leak}}-J_{\text{SERCA}}+\varepsilon \left(J_{\text{in}}-J_{\text{pm}}\right),\\ \frac{dC_{t}}{dt} & =\varepsilon (J_{\text{in}}-J_{\text{pm}}),\\ \frac{dC_{b}}{dt} & =\gamma_{1}\left(J_{\text{IPR}}-J_{\text{diff}}\right),\\ \frac{dP}{dt} & =V_{\text{plc}}-V_{\text{deg}},\\ \frac{dm_{42}^{}}{dt} & =\lambda_{m_{42}}\left(m_{42}^{\infty }-m_{42}^{}\right),\\ \frac{dh_{42}^{}}{dt} & =\lambda_{h_{42}}\left(h_{42}^{\infty }-h_{42}^{}\right), \end{array} \end{array}$$ (6)

where the choice *ε* = 0 or 1 determines whether the model is a closed-cell model or an open-cell model. We nondimensionalised the system (with *ε* = 1), not only to make the system dimensionless, but, more importantly, to identify the timescale of each variable. The nondimensionalisesd system is included in the S1 Appendix. As a result, we found that there are at least three different timescales in the system. *C*_*b*_ evolves on the fastest timescale, while *C*_*t*_ evolves on the slowest. The other variables have timescales between the fastest and the slowest.

#### The closed-cell model

When *ε* = 0, the model transforms into a closed-cell model, as there is no change in the total intracellular [Ca^2+^] (*dC*_*t*_/*dt* = 0). In order to confirm that Ca^2+^ oscillations in HSY cells indeed arise from cycles of Ca^2+^ fluxes in and out of the ER, we need to ensure that the closed-cell model, system (6) with *ε* = 0, can still produce Ca^2+^ oscillations. Then the model should be able to reproduce the Ca^2+^-free experiments in [23] and [56]. Experimentally, it is difficult to prepare a fully closed cell. However, a cell can be partially closed when placed in a Ca^2+^-free medium, thus eliminating Ca^2+^ influx from outside the cell. Tojyo et al. [23] examined the effects of Ca^2+^-free external solution on ATP-induced Ca^2+^ oscillations in HSY cells. Initially, they stimulated the cells in Ca^2+^-containing solution with ATP, and observed oscillatory responses. Then they removed extracellular Ca^2+^ and re-applied ATP. Their results showed that removing extracellular Ca^2+^ does not abolish Ca^2+^ oscillations in HSY cells, even though the cells are losing Ca^2+^ across the plasma membrane. Liu et al. [56] found a similar result in a similar experiment where HSY cells were stimulated with carbachol (CCh), and removal or re-addition of external Ca^2+^ did not terminate oscillations in [Ca^2+^]. They also showed that addition of the SERCA pump inhibitor thapsigargin abolishes CCh-induced Ca^2+^ oscillations. These experiments suggest that HSY cells do not require cycles of Ca^2+^ fluxes across the plasma membrane in order to generate Ca^2+^ oscillations. Thus, Ca^2+^ oscillations in HSY cells are primarily dependent on the periodic release and re-uptake of Ca^2+^ in the ER. Secondly, the experiments also suggest that the change in the total intracellular [Ca^2+^] is relatively slow, such that it does not much affect the whole cell behaviour. If the fluxes across the plasma membrane were fast, removing external Ca^2+^ would have resulted in termination of Ca^2+^ oscillations, with rapid loss of intracellular Ca^2+^.

## Results

### Model validation

**Fig 4:**
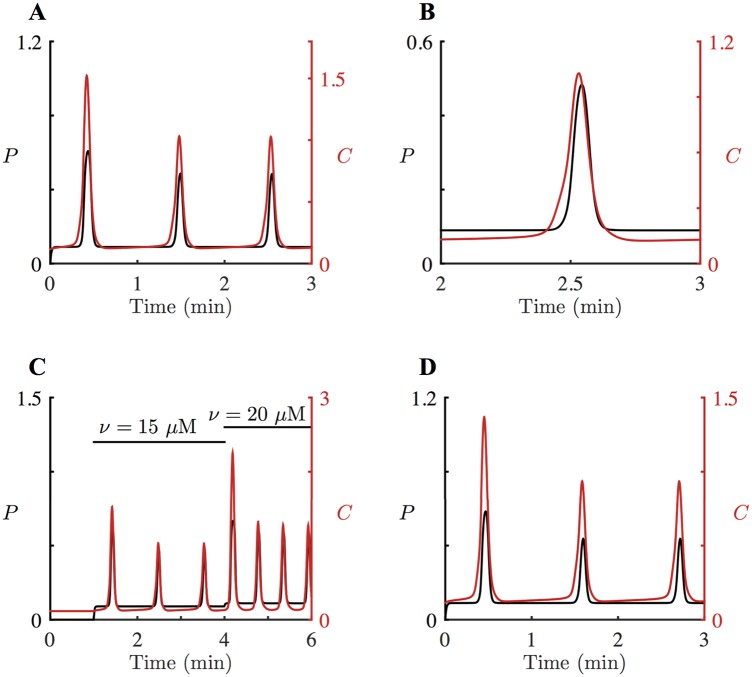
Oscillations in Ca^2+^ and IP_3_ concentrations, *C* and *P*, in the HSY cell model, system (6). **A**: When *ε* = 1, the system is stimulated with *ν* = 15 *μ*M for *t* > 0. [Ca^2+^] is shown in red; [IP_3_] is in black. The period is about 62 seconds. **B**: Enlargement of **A** for 85 < *t* < 95, showing a pair of Ca^2+^ and IP_3_ spikes. The peak of the Ca^2+^ spike is followed by the peak of the IP_3_ spike. **C**: Starting from the initial condition with *ν* = 0 *μ*M for *t* < 60 s, the system with *ε* = 1 is perturbed by *ν* = 15 *μ*M for 60 s < *t* < 240 s and *ν* = 20 *μ*M for 240 s < *t* < 360 s. The oscillations are faster at a higher agonist concentration. **D**: Coupled oscillations of [Ca^2+^] and [IP_3_] in the closed-cell model, system (6) with *ε* = 0 and *C*_*t*_ = 68 *μ*M. The system is stimulated with *ν* = 15 *μ*M for *t* > 0.

Experimentally, HSY cells were stimulated with different concentrations of ATP. Higher agonist concentrations caused faster Ca^2+^ oscillations. We model ATP application by increasing *ν*, the concentration of applied agonist. With the parameter values in Tables 1–3, the model generates oscillations of [Ca^2+^] and [IP_3_] at *ν* = 15 *μ*M (Fig 4A). Specifically, Fig 4B shows that the model captures the correct relative timing of the Ca^2+^ and IP_3_ oscillations in HSY cells, with a Ca^2+^ spike peak coming just before an IP_3_ spike peak. Furthermore, as shown in Fig 4C, the model displays faster Ca^2+^ oscillations when stronger stimulation is applied. When the system is stimulated with *ν* = 15 *μ*M, the resulting Ca^2+^ oscillations have a period of 62 seconds, while *ν* = 20 *μ*M produces oscillations with a period of 35 seconds. These model simulations are in good agreement with the experimental data in Fig 1. Also, we find that a larger *ν* causes a longer delay within a pair of [Ca^2+^] and [IP_3_] peaks. At *ν* = 15 *μ*M, a peak of [IP_3_] is about 0.63 seconds behind a [Ca^2+^] peak. This delay is extended to 0.67 seconds at *ν* = 20 *μ*M. When *ν* is decreased to 5 *μ*M or increased to 30 *μ*M, no oscillations are found.

As discussed in the previous section, experimental results suggest that plasma membrane fluxes are not necessary to produce Ca^2+^ oscillations in HSY cells [23, 56]. We simulate this by studying the closed-cell model, which can be obtained by setting *ε* = 0 in system (6) (*dC*_*t*_/*dt* = 0 *μ*M s^–1^). Physiologically, a closed cell does not have influx or efflux of Ca^2+^ across the plasma membrane, and thus maintains a constant intracellular [Ca^2+^]. For the purpose of this model simulation, *C*_*t*_ is no longer treated as a variable, but rather as a constant parameter. Thus, the closed-cell model forms a five-dimensional system, system (6) without the equation for *C*_*t*_. Preliminary analysis of the closed-cell model revealed that in order to generate Ca^2+^ oscillations in the closed-cell model, *ν* and *C*_*t*_ need to balance each other so that the system is within its oscillatory regime. In other words, even if the system is stimulated with large *ν*, the system cannot produce oscillations if *C*_*t*_ is too low. In order to generate Ca^2+^ oscillations from the closed-cell model stimulated with *ν* = 15 *μ*M, we need to set the parameter *C*_*t*_ to a value between 65 *μ*M and 78 *μ*M.

Fig 4D shows the model simulation of the closed-cell model, with a fixed *C*_*t*_ = 68 *μ*M. At *t* = 0 s, the system is at its steady state without any stimulation; agonist is then applied with *ν* = 15 *μ*M for *t* > 0. This shows that the closed-cell model can exhibit Ca^2+^ oscillations, which agrees with the experimental data that suggest Ca^2+^ oscillations in HSY cells do not require fluxes across the plasma membrane.

Thus, our model suggests that IP_3_ oscillations in HSY cells do not seem to be essential in generating Ca^2+^ oscillations. We hypothesise that the main mechanism for generating Ca^2+^ oscillations in HSY cells is Ca^2+^ feedback on IPR. We aim to validate this hypothesis through proposing model predictions, and comparing them to experimental data.

### Model predictions and experimental verification

#### Ca^2+^-free medium

The Ca^2+^-free medium experiment in Tojyo et al. [23] stimulated HSY cells with ATP, which generated Ca^2+^ oscillations, and repeatedly interchanged external solutions between Ca^2+^-containing and Ca^2+^-free mediums. Similarly, Liu et al. [56] used CCh to stimulate HSY cells, and studied the Ca^2+^ response to removal and re-addition of external Ca^2+^. However, the question of long-term behaviour in Ca^2+^-free conditions was not addressed in those experiments. We thus study this question both theoretically and experimentally.

**Fig 5:**
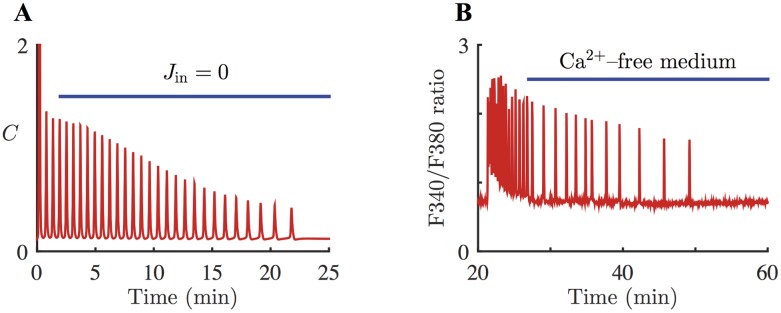
Model response to Ca^2+^-free medium and corresponding experimental data. **A**: Response of the HSY cell model, system (6) with *ε* = 1, to elimination of *J*_in_. The system is stimulated with *ν* = 20 *μ*M, which generated oscillations of [Ca^2+^]. *J*_in_ is then removed from the system (*J*_in_ = 0 *μ*M s^–1^) for *t* > 120 s. As a result, the oscillations persist for about 20 mins. The oscillations show a decrease in the spike amplitude and an increase in the interspike interval after each spike. At *t* ≈ 1400 s (21 min), the system stops producing Ca^2+^ spikes. **B**: A representative Ca^2+^ response in HSY cells in a Ca^2+^-free medium. 26 out of 40 oscillating cells has a qualitatively similar response. Initially, the cells were placed in Ca^2+^-containing medium. CCh was used to generate Ca^2+^ oscillations. The external solution was changed to a Ca^2+^-free medium for the time indicated by the blue bar. The oscillations persisted for about 20 minutes in the absence of extracellular Ca^2+^.

For the model simulation of the Ca^2+^-free medium experiment, *J*_in_ is set to 0 *μ*M s^–1^ to model the situation that there is no Ca^2+^ influx from the extracellular domain into the cytosol. System (6) is stimulated with *ν* = 20 *μ*M, then *J*_in_ is turned off for *t* > 120 s. Fig 5A shows the corresponding model simulation. As explained before, our model has slow Ca^2+^ fluxes across the plasma membrane. Thus, when *J*_in_ is removed at *t* = 120 s, the model behaves much like a closed-cell model for a transient period. The model predicts that removing *J*_in_ does not stop the Ca^2+^ oscillations immediately. Physiologically speaking, this means that there is enough Ca^2+^ in the ER to fire Ca^2+^ spikes, even without any Ca^2+^ contribution from outside of the cell. However, the spike amplitude decreases and the interspike interval gets longer after each spike until the oscillations terminate eventually. *C*_*t*_ is a slow variable in the system, and it decreases slowly over time to the point where the system is no longer in the oscillatory range. With *J*_in_ = 0, the rate of change for *C*_*t*_ becomes negative (*dC_t_*/*dt* = −*J*_pm_ < 0). Consequently, *C*_*t*_ in the model eventually decreases to 0 *μ*M, which indicates that the cell becomes completely depleted in Ca^2+^.

A representative experimental trace of [Ca^2+^] in a HSY cell that was placed in Ca^2+^-free solution is shown in Fig 5B. Initially, there was free Ca^2+^ in the extracellular domain, as the cells were surrounded by Ca^2+^-containing medium. CCh was used to stimulate the cells and induce Ca^2+^ oscillations. After some time, the cells were perfused with Ca^2+^-free solution, to wash away all the extracellular Ca^2+^. As we predicted from the model, Ca^2+^ spikes persisted for a while, before they eventually stopped. 26 out of 40 oscillating cells showed similar responses. In particular, the model predicts that it takes about 20 minutes for the oscillations to disappear, which is confirmed by the representative response in Fig 5B. Over the time, the cell must have been losing Ca^2+^ across the plasma membrane, until eventually intracellular [Ca^2+^] was too low to allow another Ca^2+^ spike. Also, in the absence of the external Ca^2+^, each interspike interval was longer than the previous one, as predicted by the model.

#### IP_3_ pulses

The [IP_3_] in the cytosol affects the open probability of the IPR, and hence governs the majority of Ca^2+^ release from the ER. In order to investigate the response of intracellular [Ca^2+^] to an increase of [IP_3_], experimentalists often use caged IP_3_, which is biologically inactive, and then release IP_3_ by flashing UV light. This photoreleased IP_3_, which has the same function as IP_3_, binds to the IPR and opens them. However, photoreleased IP_3_ metabolises more slowly than IP_3_, and therefore stays longer in a cell [57].

We model the release of caged IP_3_ by adding another variable that represents the concentration of photoreleased IP_3_, denoted by *P*_s_. Since photoreleased IP_3_ behaves like IP_3_, every *P* in the IPR model and in *J*_ROCC_ in system (6) is rewritten as *P* + *P*_s_, in order to include the effect of photoreleased IP_3_ on IPR and ROCC. Experimentally, a flash of UV light causes a sharp rise in the concentration of photoreleased IP_3_. Based on this, the production rate of photoreleased IP_3_ is modeled as in [58],

$$V_{s\_\text{plc}}\left(t\right)=MH\left(t-t_{0}\right)H\left(t_{0}+△-t\right),$$ (7)

where *H* is the Heaviside function

$$\begin{array}{c} H\left(t-t_{0}\right)=\left\{\begin{array}{cc} 0 & \text{if}\;t\leq t_{0},\\ 1 & \text{if}\;t>t_{0}, \end{array}\right. \end{array}$$

*M* is the pulse magnitude, *t*_0_ is the time at which the pulse starts and △ is the pulse duration. The production rate of photoreleased IP_3_ for *t* < *t*_0_ and *t* > *t*_0_ + △ is 0 *μ*M s^–1^. Similarly to IP_3_, we assume a constant degradation rate of photoreleased IP_3_,

$$V_{s\_\text{deg}}=r_{s\_\text{deg}}P_{s}.$$ (8)

However, since photoreleased IP_3_ metabolises more slowly than IP_3_, we assume that *r*_s_deg_ < *r*_deg_. The equation for *P*_s_ is

$$\frac{dP_{s}}{dt}=V_{s\_\text{plc}}-V_{s\_\text{deg}}.$$ (9)

**Fig 6:**
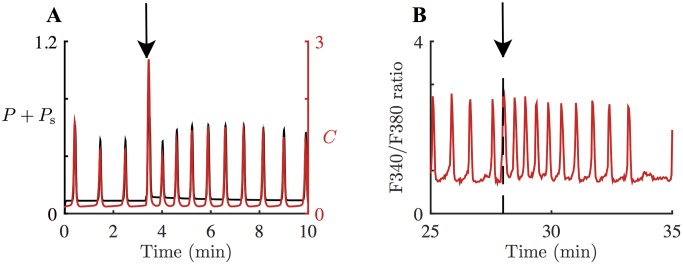
Model response to a pulse of photoreleased [IP_3_] and corresponding experimental data. **A**: Response of the HSY cell model, system (6) with *ε* = 1, to a pulse of *P*_s_, Eq (9). The system is stimulated with *ν* = 15 *μ*M at *t* = 0 s. The pulse is applied at *t* = 200 s, indicated by the arrow. Ca^2+^ concentration, *C*, is in red; the total IP_3_ concentration, including photoreleased [IP_3_], is in black. The pulse increases the frequency of the Ca^2+^ oscillations by 84%. As photoreleased IP_3_ degrades slower than IP_3_, the oscillations return only slowly to the original frequency. **B**: A representative Ca^2+^ response to photolysis of caged IP_3_ in HSY cells. 20 out of 26 oscillating cells had a qualitatively similar response. The cells were prepared with ATP and caged IP_3_, and then a flash of UV light was applied at the time indicated by the arrow and the dashed line. The cells exhibited an increase in their oscillation frequencies. The frequency increase in the representative cell was about 85%.

Fig 6A shows a model simulation with *ν* = 15 *μ*M, *t*_0_ = 200 s, *M* = 0.04 *μ*M s^–1^, △ = 0.7 s and *r*_s_deg_ = 0.006 s^–1^. The black curve in the figure represents the sum of *P* and *P*_s_, the total effective concentration of IP_3_. A pulse of *P*_s_ increases the rate of the following series of Ca^2+^ spikes. As *P*_s_ gradually decreases, the system slowly recovers its former Ca^2+^ spiking rate. In this model simulation, the pulse causes an 84% increase in the oscillation frequency.

**Fig 7:**
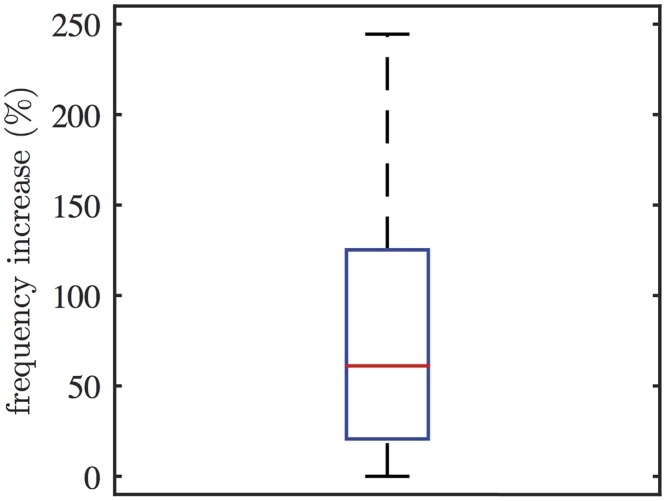
Changes in oscillation frequency induced by the photolysis of caged IP_3_. Photolysis of caged IP_3_ induced a transient increase in the oscillation frequency (20 out of 26 oscillating cells). For each cell, the oscillation frequencies before and after the photolysis were measured for comparison. On average, the photolysis increased the oscillation frequency by 77%.

Fig 6B shows a representative Ca^2+^ response to photolysis of caged IP_3_ in HSY cells. The cells were initially stimulated with ATP, and then treated with caged IP_3_ to generate Ca^2+^ oscillations. The cells were exposed to a flash of UV light, while they were actively firing Ca^2+^ spikes. The experimental traces show that a sudden increase in photoreleased [IP_3_] accelerated ATP-induced Ca^2+^ oscillations. However, the accelerated rate did not last long, and the oscillations slowed to the rate before the photolysis. IP_3_ concentration was not measured in this experiment; nevertheless, we expect that [IP_3_] would be oscillating as well, with its peaks preceded by [Ca^2+^] peaks. 20 out of 26 oscillating cells showed a transient increase in their oscillation frequencies in response to the photolysis of caged IP_3_. Fig 7 shows the box plot of the percentage increase in oscillation frequencies, measured from 20 cells. On average, the photolysis increased the oscillation frequency by approximately 77%. The transient increase in oscillation frequency was accurately predicted by the model.

#### Inhibition of PLC

Our next model simulations are designed to study how IP_3_ dynamics affects Ca^2+^ oscillations. As shown in Eq (4), the system has two stimuli that activate PLC: agonist and cytosolic [Ca^2+^]. The feedback of Ca^2+^ on IP_3_ production ensures coupled oscillations, which was one of the main features of the observed data in Fig 1 that we aimed to reproduce. However, because evidence suggests that IP_3_ oscillations are not necessary for Ca^2+^ oscillations to exist, we wished to study what effect the IP_3_ oscillations have, and how they affect the Ca^2+^ responses. One would expect that when positive Ca^2+^ feedback on PLC is removed, [IP_3_] no longer oscillates, as the coupling between the two is broken.

Experimentally, PLC can be inhibited by applying a PLC inhibitor, U73122. This compound has been shown to have inhibitory effects on PLC in various cell types, including smooth muscle cells [59] and pancreatic acinar cells [60]. However, U73122 is not a selective inhibitor that targets PLC activated by Ca^2+^; instead, it inhibits overall PLC, including the PLC activated by the external agonist. Thus, if cells were stimulated with external agonist, applying U73122 would terminate Ca^2+^ oscillations as there is no other way to produce IP_3_. In order to overcome this constraint, we use caged IP_3_ to introduce photoreleased IP_3_ intracellularly, which is independent of PLC. Continuously applying a small amount of UV light to cells that have caged IP_3_ produces photoreleased IP_3_ at a constant rate. As the degradation rate of photoreleased IP_3_ is also constant, the concentration of photoreleased IP_3_ is expected to stabilise at some steady state concentration. Thus, the cells now have a constant photoreleased [IP_3_] that can induce Ca^2+^ oscillations, without any contribution from PLC. In addition, some studies show that U73122 is not a selective inhibitor of PLC, and that it has other effects, independent of its effect on PLC [61–63]. Based on evidence that U73122 inhibits SERCA pumps, the model simulation includes this effect as well [64].

To model a constant production rate of photoreleased IP_3_ from low-level continuous UV light, we use Eq (9) with a constant *V*_S_plc_,

$$V_{s\_\text{plc}}=k_{s\_\text{plc}}.$$

The equation for photoreleased [IP_3_] (*P*_s_) is now

$$\frac{dP_{s}}{dt}=k_{s\_\text{plc}}-r_{s\_\text{deg}}P_{s},$$ (10)

which has a steady state at $\bar{P_{s}}=\frac{k_{s\_\text{plc}}}{r_{s\_\text{deg}}}$. In the model simulations, *k*_s_plc_ needs to be large enough to bring *P*_s_ above the minimum required for Ca^2+^ oscillations. When the Ca^2+^ concentration exceeds the PLC activation threshold, positive feedback on PLC in Eq (4) comes into play and starts generating IP_3_ oscillations. We then remove Ca^2+^ feedback on PLC, by decreasing the coupling strength between Ca^2+^ and IP_3_, *ψ*_2_. Alternatively, we can increase *K*_plc_, which would have the same effect on the model. As *ψ*_2_ decreases, the term $\psi_{2}\frac{C^{4}}{C^{4}+K_{\text{plc}}^{4}}$ gets close to 0, thus decreasing the effect that Ca^2+^ has on IP_3_ production. Additionally, we take into account that the PLC inhibitor could also block SERCA pumps; we model this by decreasing the maximum capacity flux of SERCA, *V*_S_.

**Fig 8:**
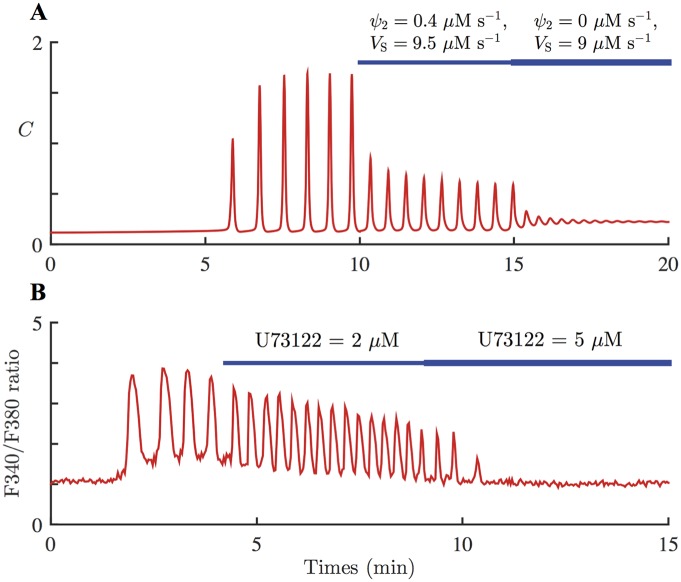
Model response to inhibition of PLC and corresponding experimental data. **A**: Response of the HSY cell model, system (6) with *ε* = 1, to the inhibition of positive Ca^2+^ feedback on IP_3_, as well as SERCA pumps. The system is stimulated with slowly increasing *P*_s_, Eq (10) with *k*_s_plc_ = 0.0006 *μ*M s^–1^. At *t* = 600 s, the magnitude of the Ca^2+^ feedback on PLC is halved, and the maximum SERCA capacity is also decreased. The oscillation frequency increases by 22%. At *t* = 900 s, the coupling between Ca^2+^ and IP_3_ is completely removed, and the SERCA capacity is decreased further. **B**: A representative Ca^2+^ response to injection of U73122 in HSY cells. 13 out of 21 oscillating cells has a qualitatively similar response. The cells were prepared with caged IP_3_ and continuously exposed to low-level UV light. photoreleased IP_3_ then generated Ca^2+^ oscillations. 2 *μ*M of U73122 was applied to the cells for the time indicated by the thin blue bar, then the dose was increased to 5 *μ*M for the time indicated by the thicker blue bar. The addition of U73122 increased the oscillation frequency. The frequency increase in the representative cell was about 46%.

Fig 8A shows the model simulation of applying continuous UV light, and then adding PLC inhibitor to remove Ca^2+^ activation on PLC. The system is stimulated with *k*_s_plc_ = 0.0006 *μ*M s^–1^, without any external agonist (*ν* = 0 *μ*M). Photoreleased [IP_3_] slowly accumulates to its steady state concentration, $\bar{P_{s}}=0.1$ *μ*M. Once there is enough photoreleased IP_3_ and Ca^2+^, the system starts generating Ca^2+^ spikes (at *t* ≈ 350 s). At *t* = 600 s, *ψ*_2_ is decreased to 0.4 *μ*M s^–1^ which halves the coupling strength between Ca^2+^ and IP_3_, and *V*_S_ is decreased to 9.5 *μ*M s^–1^. The model predicts that the effects of PLC inhibition are a decrease in the Ca^2+^ spike amplitude and an increase in the oscillation frequency. The frequency increase in the model simulation shown in Fig 8A is about 22%. However, numerical computations indicate that the long-term increase in the oscillation frequency is about 18%; see Table 4. At *t* = 900 s, *ψ*_2_ and *V*_S_ are further decreased to 0 *μ*M s^–1^ and 9 *μ*M s^–1^, respectively, to amplify the effects of applying a PLC inhibitor, and eliminate Ca^2+^ feedback on PLC. As a results, the oscillations are almost destroyed. Table 4 shows the periods of the stable periodic orbits generated from system (6), with different combinations of parameter values for *ψ*_2_ and *V*_S_. It is clear that diminishing Ca^2+^ feedback on PLC alone induces a substantial decrease in the oscillation period. When only *V*_S_ is decreased, the oscillations get slower, but the impact on the oscillation period is greater when both *ψ*_2_ and *V*_S_ are decreased.

**Table 4:** Periods of the long-term oscillations in system (6), with different values of *ψ*_2_ and *V*_S_.

UV	*ψ*_2_	*V*_S_	Oscillation period
0.0006 *μ*M s^–1^	0.8 *μ*M s^–1^	10 *μ*M s^–1^	52.24 seconds
0.0006 *μ*M s^–1^	0.8 *μ*M s^–1^	9.5 *μ*M s^–1^	54.09 seconds
0.0006 *μ*M s^–1^	0.4 *μ*M s^–1^	10 *μ*M s^–1^	45.51 seconds
0.0006 *μ*M s^–1^	0.4 *μ*M s^–1^	9.5 *μ*M s^–1^	44.22 seconds
0.0006 *μ*M s^–1^	0 *μ*M s^–1^	10 *μ*M s^–1^	42.06 seconds

**Fig 9:**
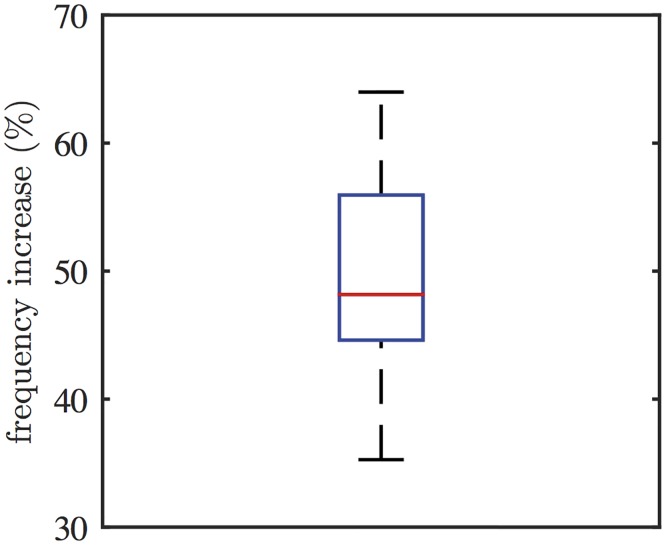
Changes in oscillation frequency induced by the addition of 2 *μ*M of U73122. 13 out of 21 oscillating cells showed an increase in oscillation frequency. For each cell, the frequencies before and after the addition of U73122 were measured for comparison. On average, applying 2 *μ*M of U73122 increased the oscillation frequency by about 50%.

The experimental test of this model prediction is shown in Fig 8B, which shows a representative Ca^2+^ response in an HSY cell to the PLC inhibitor U73122. 13 out of 21 oscillating cells showed qualitatively similar responses. The cells were treated with caged IP_3_, and were then exposed to low UV light continuously. As a result, photoreleased IP_3_ generated Ca^2+^ oscillations. The cells were then subjected to 2 *μ*M of U73122 to inhibit PLC. Subsequently, the dose of U73122 was increased to 5 *μ*M to enhance the effect of U73122. The result shows that impeding PLC decreased the amplitude of the Ca^2+^ spikes, while increasing their frequency. When a higher concentration of U73122 was applied, the oscillations were abolished. These results verify the model prediction. Fig 9 shows the box plot of the percentage increases in oscillation frequency induced by the addition of 2 *μ*M of U73122, measured from 13 cells. On average, the cells exhibited a 50% increase in the oscillation frequency.

**Fig 10:**
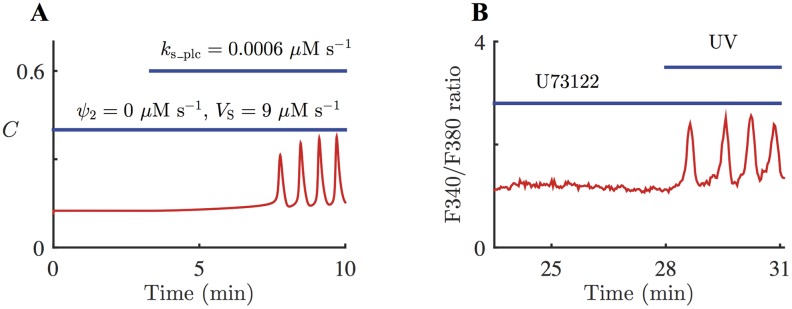
Model response to continuous photolysis of caged IP_3_, after the inhibition of PLC, and corresponding experimental data. **A**: Response of the HSY cell model, system (6) with *ε* = 1, to the addition of *P*_s_, Eq (10), while Ca^2+^ feedback on PLC is completely inhibited. System (6) is simulated with *ψ*_2_ = 0 *μ*M s^–1^ and *V*_S_ = 9 *μ*M s^–1^, to include the effects of the addition of U73122, indicated by the lower blue bar. From *t* = 200 s, *k*_s_plc_ is increased to 0.0006 *μ*M s^–1^, to model the continuous photolysis of caged IP_3_, indicated by the upper blue line. The system starts generating Ca^2+^ spikes at around *t* = 500 s. **B**: A representative Ca^2+^ response to the addition of U73122, followed by photolysis of caged IP_3_. 13 out of 20 oscillating cells has a qualitatively similar response. The cells were prepared with caged IP_3_ and U73122, and then exposed to UV light during the time indicated by the upper blue bar.

With this model, we can also investigate the effects of the photolysis of caged IP_3_, while PLC activity is being inhibited. Fig 10A shows the model simulation of the addition of U73122, followed by continuous UV light. Initially, system (6) with Eq (10) is simulated with *ε* = 1, *ψ*_2_ = 0 *μ*M s^–1^, and *V*_S_ = 9 *μ*M s^–1^ to model the addition of U73122. At *t* = 200 s, *k*_s_plc_ is increased to 0.00056 *μ*M s^–1^ to describe a constant increase in *P*_s_, which mimics the application of continuous UV light after the inhibition of PLC. As a result, the system starts generating Ca^2+^ spikes at around *t* = 500 s. The model predicts that photolysis of caged IP_3_ can elicit Ca^2+^ oscillations in HSY cells, even under inhibited PLC activity.

This model prediction was tested through experiments, with the result shown in Fig 10B. Initially, 20 HSY cells were prepared with caged IP_3_. Then they were applied with U73122, in order to inhibit any PLC activities. Subsequently, they were continuously exposed to UV light for the photolysis of caged IP_3_ at a constant rate. As a result, 13 cells exhibited a series of Ca^2+^ spikes, which indicates that Ca^2+^ oscillations can be generated from photoreleased IP_3_, without any contribution from PLC activities. The data show that the oscillations emerged as soon as the photolysis took place, whereas in the model simulation, it takes about 5 mins before the first oscillation is generated. This discrepancy is addressed in more detail in the Discussion.

**Fig 11:**
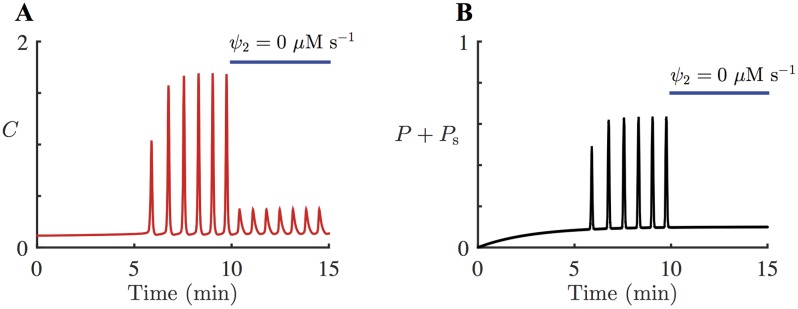
Model simulations showing Ca^2+^ and IP_3_ responses to inhibition of Ca^2+^ feedback on PLC. System (6) with *ε* = 1 is stimulated with slowly increasing *P*_s_, Eq (10) with *k*_s_plc_ = 0.0006 *μ*M s^–1^. At *t* = 600 s, *ψ*_2_ is decreased to 0 *μ*M s^–1^ to completely break the coupling between Ca^2+^ and IP_3_. All the other parameters are unchanged. The resulting oscillations have a smaller amplitude and a higher frequency. The change in the oscillation frequency is not obvious in this figure. However, numerical computations indicate that when *ψ*_2_ is decreased from 0.8 *μ*M s^–1^ to 0 *μ*M s^–1^, the increase in the frequency is about 24%; see Table 4. **A**: The red traces show the Ca^2+^ response in the model simulation. **B**: The black traces show the sum of [IP_3_] and photoreleased [IP_3_]. Without any Ca^2+^ feedback on PLC, [IP_3_] no longer oscillates, and the total effective concentration of IP_3_ stabilises at a constant level.

As U73122 inhibits both PLC and SERCA, it is difficult to discern the effects of inhibiting PLC only, independent of SERCA activities. However, it can be studied through model simulations. Fig 11 shows the model simulation of completely removing Ca^2+^ feedback on PLC. From *t* = 0 s, the system is stimulated with *k*_s_plc_ = 0.0006 *μ*M s^–1^, which induces Ca^2+^ oscillations at around *t* = 300 s. At *t* = 600s, *ψ*_2_ is decreased from 0.8 *μ*M s^–1^ to 0 *μ*M s^–1^, to simulate the complete inhibition of positive Ca^2+^ feedback on PLC. In this simulation *ψ*_2_ is decreased to 0 *μ*M s^–1^ while all the other parameters are kept the same. Setting *ψ*_2_ = 0 *μ*M s^–1^ essentially removes the coupling between Ca^2+^ and IP_3_. Without any PLC activation from Ca^2+^ or external agonist, [IP_3_] decreases to 0 *μ*M. This allows the total cytosolic [IP_3_], which is the sum of [IP_3_] and photoreleased [IP_3_], to relax to its steady state, $\bar{P_{s}}=0.1$ *μ*M; see Fig 11B. The numerical computations indicate that when the coupling between Ca^2+^ and IP_3_ is completely eliminated, the oscillation frequency increases by about 24%; see Table 4.

## Discussion

We have constructed a mathematical model for Ca^2+^ oscillations in HSY cells, a human salivary duct cell line. The basic structure of the model follows the schematic diagram shown in Fig 2, and is based on the IPR model of [30]. In the model, the production rate of IP_3_ is dependent on *ν*, the concentration of applied agonist, and cytosolic [Ca^2+^]. As shown in Eq (4), we expressed Ca^2+^ feedback on the production of IP_3_ as a Hill function, where the parameter *K*_plc_ is the PLC activation constant. The parameters in Tables 1–3 were chosen to give good agreement with the experimental traces of Tanimura et al. [22], where they observed coupled oscillations of [Ca^2+^] and [IP_3_] in HSY cells, with IP_3_ spike peaks preceded by Ca^2+^ spike peaks (see Fig 1). Additionally, Tojyo et al. [23] and Liu et al. [56] demonstrated that oscillations in [Ca^2+^] in HSY cells are independent of Ca^2+^ influx, suggesting that the release and re-uptake of Ca^2+^ in the ER are sufficient to generate oscillations. As shown in Fig 4, our HSY cell model reproduced the coupled oscillations with the correct order of the peaks, and the closed-cell model also exhibited oscillations.

We then used the model to make predictions about Ca^2+^ responses to three different experimental procedures: 1) elimination of extracellular Ca^2+^, 2) photolysis of caged IP_3_, and 3) inhibition of PLC. Experiments were conducted to test the model predictions. Firstly, the model predicted that when external Ca^2+^ is removed, intracellular [Ca^2+^] keeps oscillating but with slowly decreasing frequency. The second model simulation predicted that a pulse of photoreleased [IP_3_] accelerates Ca^2+^ oscillations. In the third simulation, we wanted to investigate the role of the coupling between Ca^2+^ and IP_3_, particularly whether it is essential for Ca^2+^ oscillations in HSY cells. We generated Ca^2+^ oscillations using photoreleased IP_3_, which induced IP_3_ oscillations through positive Ca^2+^ feedback on IP_3_. We then removed Ca^2+^ activation on the production of IP_3_, while photoreleased IP_3_ stayed unaffected. The model simulation showed that the Ca^2+^ oscillations persist at a constant concentration of photoreleased IP_3_, in the absence of oscillating [IP_3_]. The result corroborates a hypothesis of Tanimura et al. [22], where it was conjectured that IP_3_ oscillations are not necessary to generate Ca^2+^ oscillations in HSY cells. Thus, the model confirms that it is a Class I mechanism that gives rise to Ca^2+^ oscillations in HSY cells, and confirms also that the IP_3_ oscillations accompanying the Ca^2+^ oscillations, although not required, serve to modulate the oscillation period.

### Quantitative variation

Our model is a deterministic model, which assumes that IPR clusters are continuously distributed per unit cell volume, and that they behave synchronously: either all open or all closed. Also, the frequency and the amplitude of Ca^2+^ oscillations are pre-determined by the model parameters. However, the experimental data exhibit stochastic Ca^2+^ events, with spontaneous spikes that vary in frequency and amplitude. They suggest that the number of clusters with open IPR for each Ca^2+^ spike is determined through a stochastic process. In a stochastic model, the number of IPR involved in the spike initiation and the interspike interval form distributions, and random Ca^2+^ release through an individual IP_3_ receptor plays an important role in generating global Ca^2+^ signals.

However, Cao et al. [30] compared the Ca^2+^ dynamics in a model with stochastic kinetics of the receptors with that of a model with deterministic receptor kinetics and showed that deterministic models make qualitatively accurate predictions about whole-cell Ca^2+^ dynamics. We follow Cao et al. in believing that our deterministic model is sufficient to study qualitative features of Ca^2+^ dynamics in HSY cells. Introducing stochastic components to the model may reduce the quantitative discrepancies between the model results and the experimental data, but would require far more extensive and difficult computations.

The model consists of a set of ODEs, and hence assumes homogeneous cytosolic [Ca^2+^] and [IP_3_]. In other words, Ca^2+^ spikes in the model represent global and simultaneous increase and decrease in the cytosolic [Ca^2+^]. Practically, this is an incorrect assumption, as it does not take into account that the cytosol is spatially inhomogeneous. When the ER starts to release Ca^2+^, the [Ca^2+^] near a dense group of IPR clusters is unlikely to be the same as in some other parts of the cytosol, where the clusters are sparse, particularly since Ca^2+^ diffusion is relatively slow. Mathematically, we can incorporate spatial dimensions in our ODE model by extending it to a partial differential equation (PDE) model, and reproduce Ca^2+^ waves across the domain. With a PDE model, we can specify the spatial distribution of the receptor clusters, and hence we can include coupled reactions between the neighbouring clusters. For instance, a small Ca^2+^ release from a cluster can trigger a series of releases from the adjacent clusters, and consequently, lead to global Ca^2+^ waves. In order to build a Ca^2+^ model with PDEs, we need high resolution images of intracellular Ca^2+^ dynamics, so that we can observe Ca^2+^ waves and accurately analyse the wave speed, direction, and amplitude. At this stage, no such data are available from HSY cells, making it difficult to construct a quantitative version of such a model. Thus, although it is possible that spatial aspects might explain some of the quantitative differences between the present model and the data, we cannot be entirely confident in the ability of a spatial model to do so.

Numerous studies have reported the possibility of mitochondria serving as a Ca^2+^ buffer [65–67]. Given the fact that mitochondrial Ca^2+^ uptake sites can be localised near Ca^2+^ release channels, it is possible that mitochondria could influence the rate of increase and the amplitude of a Ca^2+^ spike during agonist-induced Ca^2+^ oscillations. Also, mitochondria potentially increase the time it takes for a Ca^2+^ spike to reach its baseline [Ca^2+^] from the peak, as mitochondria release Ca^2+^ back into the cytosol during the decay phase of the spike. However, there is not enough information about the regulation of mitochondria in HSY cells for us to implement it in our model.

### Activation kinetics of the IPR

The original IPR model of Cao et al. [30] was used to explain Ca^2+^ oscillations in airway smooth muscle cells, which exhibit fast oscillations, with periods on the order of a few seconds. Although many of the parameters of the IPR model were determined by fitting to single-channel data, not all of the transition rates could be determined from the data. In particular, λ_*h*_42__, the rate at which *h*_42_ responds to changes in [Ca^2+^], was not determined by the single-channel data, and thus *L* and *H* were chosen by Cao et al. so as to give a model that could reproduce the fast Ca^2+^ oscillations in airway smooth muscle cells. However, there is no reason to believe that these parameters (which are essentially phenomenological, rather than based directly on known biophysical processes) should be the same in HSY cells. Thus, based on the slow timescale of Ca^2+^ oscillations in HSY cells, we assume that *L* and *H* are smaller in HSY cells than in airway smooth muscle cells. Extensive efforts to reproduce the long periods seen in HSY cells by varying other model parameters were unsuccessful; although the period can be changed a small amount by changes in other parameters, changes in *L* and *H* are by far the most effective at doing so.

In effect, we are predicting that the period of Ca^2+^ oscillations can be most effectively manipulated by changing how fast the rate of IPR activation by Ca^2+^ responds to changes in [Ca^2+^]. However, we have not tested this prediction directly, and so this proposed mechanism for generating slow oscillations in HSY cells remain hypothetical.

### Modeling continuous photolysis of caged IP_3_

In order to study the effects of Ca^2+^-activated PLC on Ca^2+^ oscillations in HSY cells, a PLC inhibitor, U73122, was applied to the cells. For the purpose of this experiment, the cells were pre-treated with caged IP_3_, so that their Ca^2+^ oscillations could be initiated by photoreleased IP_3_, in a PLC-independent manner. According to our model simulations, it takes a certain amount of time (about 5 mins) to trigger Ca^2+^ oscillations from the slowly increasing photoreleased [IP_3_]; see Figs 8A and 10A. On the other hand, the corresponding experimental data suggest that the continuous photolysis of caged IP_3_ almost immediately induces Ca^2+^ spikes, as shown in Figs 8B and 10B. We suspect that this discrepancy is caused by the simplification in our method of modeling the continuous photolysis of caged IP_3_. Our model assumes that the continuous photolysis of caged IP_3_ would initially give a constant increase in photoreleased [IP_3_], and hence would be equivalent to having constant rates of photoreleased IP_3_ production and degradation. However, the actual biological process may be quite different from our model assumption. For instance, at the beginning of the photolysis, there could be a sharp increase in photoreleased [IP_3_], followed by a decrease to a saturated level. This type of reaction would explain the immediate Ca^2+^ responses to the continuous photolysis of caged IP_3_.

Although there is still room for improvement in modeling the continuous photolysis of caged IP_3_ with the correct timescale, our model accurately predicts that the photolysis can trigger Ca^2+^ oscillations, even when PLC is inhibited. Furthermore, it is not our main purpose to model the accurate biological process of the continuous photolysis. Thus, we are not concerned about the time that it takes for the model to generate Ca^2+^ oscillations with constant rates of photoreleased IP_3_ production and degradation.

### Ca^2+^ feedback on PLC

The experimental data from Tanimura et al. [22] shows that there are coupled oscillations of [Ca^2+^] and [IP_3_] in HSY cells, which suggests the inclusion of positive and/or negative feedback in the model. Specifically, peaks of IP_3_ spikes being preceded by those of Ca^2+^ spikes strongly suggests the presence of positive Ca^2+^ feedback on the formation of IP_3_. Dupont et al. [35] pointed out that Ca^2+^ feedback on IP_3_ degradation could explain Ca^2+^ oscillations with relatively low frequency. In this case, each Ca^2+^ spike would cause a subsequent decrease in [IP_3_], and there would be a latency before the next Ca^2+^ spike as [IP_3_] would need to build up to a certain level to activate the IPR again. We studied a case where both positive and negative feedback coexist in the model. For this case, both production and degradation rates of IP_3_ were modeled as functions of [Ca^2+^]. The main conflict between the model with positive and negative feedback and the data from [22] was that the model could not reproduce the order of [Ca^2+^] and [IP_3_] peaks. In fact, the model generated coupled oscillations with a IP_3_ spike peak occurring just before a Ca^2+^ spike peak, which is not the case for HSY cells. For this reason, we decided not to include negative feedback in the model. However, we note that the phase shift in peak order could be relevant to other cellular systems in which Ca^2+^ feedback on the degradation of IP_3_ leads to a system with negative feedback, such as the models of Meyer and Stryer [31] and Dupont and Erneux [68].

Nezu et al. [53] observed Ca^2+^ and IP_3_ responses in HSY cells, induced by mechanical stimulation. Their results suggested the existence of a family of PLCs in HSY cells that is independent of external agonist such as ATP and CCh. We formulated three different equations for the production rate of IP_3_:

(a) PLC by agonist + PLC by Ca^2+^
(b) PLC by agonist + PLC by agonist and Ca^2+^ + PLC by Ca^2+^
(c) PLC by agonist and Ca^2+^ + PLC by Ca^2+^

For cases (b) and (c), we assumed that PLC that gets activated by both agonist and Ca^2+^ is expressed as a product of some functions, *f*(*ν*) ⋅ *g*(*C*). If we express it as a sum, *f*(*ν*) + *g*(*C*), the structure of the PLC equations for these cases would not be any different from that of case (a). We studied the model responses with each expression, and observed that they showed no qualitative difference. Our model simulations show that case (a), which is the simplest form of all three, correctly predicts Ca^2+^ responses in all the experimental settings.

Positive and negative Ca^2+^ feedback on IP_3_ was extensively studied in Politi et al. [32] and Gaspers et al. [33], both theoretically and experimentally; they observed qualitatively different behaviours in oscillations. Politi et al. built two different Ca^2+^ models, one with Ca^2+^ activation on PLC (positive feedback), and the other with Ca^2+^ activation on IP_3_K (negative feedback). Both models exhibited Ca^2+^ oscillations with sharp spikes from the basal Ca^2+^ level. However, the shapes of the IP_3_ oscillations in the models were different. In the positive feedback model, the shape of the IP_3_ spikes was similar to that of the Ca^2+^ spike, with a sharp rise from the basal line to the peak and a fast decrease down to the basal level. On the other hand, IP_3_ oscillations in the negative feedback model had a zig-zag pattern, where a Ca^2+^ spike caused a sudden decrease in [IP_3_], followed by a slow increase until the next Ca^2+^ spike. Politi et al. also found that both positive and negative Ca^2+^ feedback on IP_3_ regulation extend the range of oscillation frequencies.

Gaspers et al. compared the effects of introducing an IP_3_ buffer in models with positive or negative Ca^2+^ feedback on IP_3_. In the absence of buffer, the models exhibited agonist-induced Ca^2+^ oscillations. When an IP_3_ buffer was added to the negative feedback model, it responded with decreased oscillation frequency and increased latency before the first spike. The oscillations persisted even at high concentrations of IP_3_ buffer. In contrast, when IP_3_ buffer was added to the positive feedback model, the oscillations were abolished. In this model, the Ca^2+^ flux across the plasma membrane was assumed to be relatively small compared to the flux across the ER membrane, and hence was neglected from the model. However, when the model had substantial Ca^2+^ fluxes across the plasma membrane, the inclusion of IP_3_ buffer slowed Ca^2+^ oscillations. They concluded that positive feedback of Ca^2+^ on the production of IP_3_ is essential for the generation of long-period, baseline-separated Ca^2+^ oscillations and waves. We have not tested the effects of introducing an IP_3_ buffer in our model. However, the results of our current work is similar to the findings of Gaspers et al., whereby positive Ca^2+^ feedback on IP_3_ regulation in HSY cells is shown to induce Ca^2+^ oscillations with lower frequency.

Bartlett et al. [34] investigated the characteristics of Ca^2+^ oscillations in hepatocytes, primarily induced by photolysis of caged IP_3_. One of their experiments involved pre-treating hepatocytes with U73122, followed by applying UV light. They observed that uncaging of IP_3_ can elicit oscillatory Ca^2+^ behaviours even after PLC is inhibited. Based on their experimental results, they concluded that Ca^2+^ oscillations induced by uncaging of IP_3_ in hepatocytes (in the absence of external stimulation) do not require PLC activities, and are generated from CICR. Also, their data indicated that positive Ca^2+^ feedback on PLC is not a key player in Ca^2+^ oscillations elicited by photolysis of caged IP_3_. Our experiments and model simulations of intracellular Ca^2+^ dynamics in HSY cells lead to observations that are parallel to those of Bartlett et al., where both HSY cells and hepatocytes show oscillatory Ca^2+^ responses to photolysis of caged IP_3_, that seem to be independent of PLC activation. We then further investigated the contribution of Ca^2+^ activation on PLC to caged IP_3_-induced oscillations. The model simulations revealed that although positive Ca^2+^ feedback on PLC may not be necessary to trigger the oscillations in HSY cells, it has some effects on the oscillation frequency. It would be interesting to test whether Ca^2+^ activation on PLC in hepatocytes has the same effects on the oscillations generated from photolysis of caged IP_3_, without any external stimulation.

### PLC inhibition and the frequency of Ca^2+^ oscillations

We wanted to study Ca^2+^ oscillations without Ca^2+^ feedback on PLC. Mathematically, this can be achieved by decreasing the parameter *ψ*_2_ from 0.8 *μ*M s^–1^ to a smaller value, so that the role of Ca^2+^ on PLC is minimised. However, once cells are stimulated with external agonist, we cannot selectively inhibit Ca^2+^ feedback on PLC, as U73122 inhibits overall PLC, including agonist-activated PLC. It was necessary for the model to have Ca^2+^ oscillations in the absence of external agonist, as a simple decrease of *ψ*_2_ alone would prevent the formation of IP_3_, thus preventing any oscillatory activity. We thus simulated the situation where *ψ*_2_ is decreased at the same time as IP_3_ is photoreleased by continuous application of low-level UV light. This lets us predict the Ca^2+^ responses under the conditions where PLC is not being stimulated by Ca^2+^, but with photoreleased IP_3_ in the background as a primary stimulus of the oscillations.

When HSY cells are pre-treated with caged IP_3_ and exposed to continuous UV light, the concentration of photoreleased IP_3_ is expected to reach a steady state, due to the constant rates of production and degradation. During Ca^2+^ oscillations, additional IP_3_ is introduced on top of the baseline level of photoreleased IP_3_. Thus, the average concentration of IP_3_ during Ca^2+^ oscillations is lower when Ca^2+^-activated PLC is eliminated. In many cell types, it is generally the case that, within an appropriate range, higher concentrations of IP_3_ lead to faster oscillations. However, the model simulation of inhibiting PLC while simultaneously photo-releasing IP_3_, showed an increase in oscillation frequency. Although this result seems somewhat counterintuitive from a biophysical point of view, it is in fact consistent with the theory of oscillations that are generated by positive feedback. Tsai et al. [69] studied the effects of positive feedback in a number of biophysical models that contain both positive and negative feedback mechanisms. One of their main conclusions is that ranges of oscillation frequency are wider when the models have stronger positive feedback, and that an increase in the strength of the positive feedback will commonly lead to a decrease in oscillation frequency.

**Fig 12:**
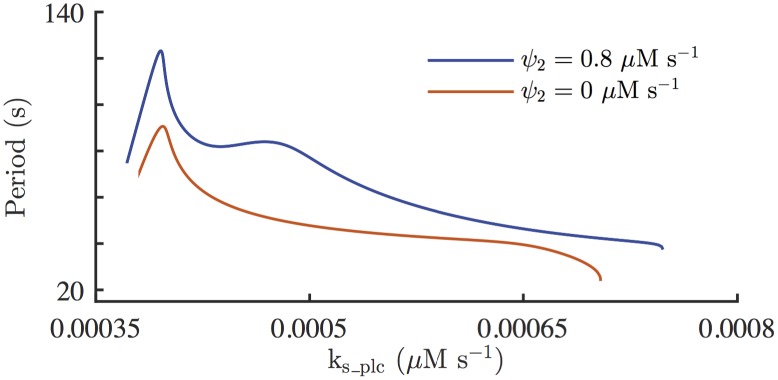
Periods of Ca^2+^ oscillations in the HSY cell model, system (6) with *ε* = 1 and two different values of *ψ*_2_. The blue and red curves show the periods of the long-term stable oscillations generated by the system with *ψ*_2_ = 0.8 *μ*M s^–1^ and 0 *μ*M s^–1^, respectively. The system is stimulated with slowly increasing *P*_s_, Eq (10). For fixed *k*_s_plc_, oscillations with *ψ*_2_ = 0 *μ*M s^–1^ have shorter period than those with *ψ*_2_ = 0.8 *μ*M s^–1^.

When we computed periods of the Ca^2+^ oscillations in the model with different *ψ*_2_ values (see Fig 12), it was clear that at a given *k*_s_plc_ (i.e., at a given production rate of photoreleased IP_3_), having larger *ψ*_2_ generates slower oscillations. For this computation, the value of parameter *V*_S_ was kept the same (*V*_S_ = 10 *μ*M s^–1^), to confirm that the elimination of the cross-coupling between Ca^2+^ and IP_3_ is responsible for the increase in oscillation frequency, without any input from modified SERCA parameters. The maximum oscillation period with *ψ*_2_ = 0.8 *μ*M s^–1^ was about 2 minutes, whereas the maximum period with *ψ*_2_ = 0 *μ*M s^–1^ was 1.5 minutes. This result agrees with what was found in Politi et al. [32], where eliminating positive Ca^2+^ feedback on PLC caused oscillations to have shorter periods. We treat Ca^2+^- and agonist-stimulated PLC as separate activities, which is different from the work of Politi et al., where Ca^2+^ activation is applied to agonist-induced PLC. However, our results resemble the findings of Politi et al., whereby the cross-coupling between Ca^2+^ and IP_3_ is shown to increase the range of oscillation frequencies. This indicates that Ca^2+^ activation on PLC in some cells, whether it is agonist-dependent or independent, may modulate oscillation frequency. Interestingly, the experimental data showed a similar result (see Fig 8B). When U73122, a PLC inhibitor, was applied to HSY cells that had oscillating [Ca^2+^], there was a clear decrease in the oscillation period and amplitude. Given the agreement between the model prediction and the experimental result, this is strong evidence that Ca^2+^ oscillations in HSY cells do not depend on simultaneous IP_3_ oscillations, but that, when IP_3_ oscillations also occur, their function is to increase the oscillation period.

## Supporting information

S1 Appendix Nondimensionalisation of the model. (PDF)

S1 Dataset HSY cells calcium fluorescence trace data. The experimental data are included as an Excel file. The name of each sheet within the file corresponds to the experimental setting. Column A’s show the number of frame and Column B’s show the corresponding time in minutes. Column D’s show the condition that was applied to the cells.
(ZIP)
