# Supplementary material for: A mathematical model of calcium dynamics in HSY cells
Source: PLoS Comput Biol. 2017 Feb 15;13(2):e1005275. doi: 10.1371/journal.pcbi.1005275 (PMC5310762; doi:10.1371/journal.pcbi.1005275)
Supplement: S1 Appendix — (PDF) [file pcbi.1005275.s001.pdf]

S1 Appendix. Nondimensionalisation of the model.
We define new dimensionless variables for (C, C_b, C_t, P, t), with

$$C = Q_C \cdot \widehat{C}, \qquad C_b = Q_C \cdot \widehat{C_b}, \qquad C_t = Q_{C_t} \cdot \widehat{C_t}, \qquad P = Q_P \cdot \widehat{P}, \qquad t = T \cdot \hat{t},$$

where Q_C, Q_{C_t}, and Q_P are typical scales of the cytosolic [Ca^{2+}], total [Ca^{2+}], and [IP_3], respectively, and T is a typical timescale. The variables m_{42} and h_{42} are already dimensionless, and their typical scales are of $O(1)$; thus, they do not need scaling. Through substitution, we derived the following dimensionless version of the system:

$$\begin{aligned}
\frac{d\widehat{C}}{d\hat{t}} &= \frac{TV_{\text{S}}}{Q_C}(\bar{J}_{\text{diff}} + \bar{J}_{\text{leak}} - \bar{J}_{\text{SERCA}}) + \frac{TV_{\text{SOCC}}}{Q_C}(\bar{J}_{\text{in}} - \bar{J}_{\text{pm}}),\\
\frac{d\widehat{C_t}}{d\hat{t}} &= \frac{TV_{\text{SOCC}}\varepsilon}{Q_{C_t}}(\bar{J}_{\text{in}} - \bar{J}_{\text{pm}}),\\
\frac{d\widehat{C_b}}{d\hat{t}} &= \frac{TV_{\text{S}}\gamma_1}{Q_C}(\bar{J}_{\text{IPR}} - \bar{J}_{\text{diff}}),\\
\frac{d\widehat{P}}{d\hat{t}} &= \frac{T\psi_2}{Q_P}(\bar{V}_{\text{plc}} - \bar{V}_{\text{deg}}),\\
\frac{dm_{42}}{d\hat{t}} &= T\lambda_{m_{42}}(m_{42}^{\infty} - m_{42}),\\
\frac{dh_{42}}{d\hat{t}} &= T\lambda_{h_{42}}(h_{42}^{\infty} - h_{42}),
\end{aligned} \tag{1}$$

with dimensionless fluxes

$$\begin{aligned}
\bar{J}_{\text{IPR}} &= \frac{k_{\text{IPR}}O_{\text{IPR}}\gamma_2}{V_{\text{S}}}\left(Q_{C_t}\widehat{C_t} - Q_C\widehat{C} - \frac{Q_C\widehat{C_b}}{\gamma_1} - \frac{Q_C\widehat{C_b}}{\gamma_2}\right),\\
\bar{J}_{\text{diff}} &= \frac{k_{\text{diff}}}{V_{\text{S}}}\left(Q_C\widehat{C_b} - Q_C\widehat{C}\right),\\
\bar{J}_{\text{leak}} &= \frac{k_{\text{leak}}\gamma_2}{V_{\text{S}}}\left(Q_{C_t}\widehat{C_t} - Q_C\widehat{C} - \frac{Q_C\widehat{C_b}}{\gamma_1} - \frac{Q_C\widehat{C}}{\gamma_2}\right),\\
\bar{J}_{\text{SERCA}} &= \frac{1}{1 + \left(\dfrac{K_{\text{S}}}{Q_C\widehat{C}}\right)^{1.75}},\\
\bar{J}_{\text{in}} &= \frac{J_{\text{leakin}}}{V_{\text{SOCC}}} + \frac{V_{\text{ROCC}}Q_P\widehat{P}}{V_{\text{SOCC}}} + \frac{1}{1 + \left(\dfrac{\gamma_2}{K_{\text{SOCC}}}\left(Q_{C_t}\widehat{C_t} - Q_C\widehat{C} - \dfrac{Q_C\widehat{C_b}}{\gamma_1}\right)\right)^4},
\end{aligned}$$

$$\bar{J}_{\text{pm}} = \frac{V_{\text{pm}}}{V_{\text{SOCC}}} \frac{1}{1 + \left(\dfrac{K_{\text{pm}}}{Q_C \widehat{C}}\right)^2},$$

$$\bar{V}_{\text{plc}} = \frac{\psi_1}{\psi_2} \frac{\nu}{K_\nu + \nu} + \frac{1}{1 + \left(\dfrac{K_{\text{plc}}}{Q_C \widehat{C}}\right)^4},$$

$$\bar{V}_{\text{deg}} = \frac{r_{\text{deg}}}{\psi_2} Q_P \widehat{P}.$$

We choose $T = Q_{C_t}/V_{\text{SOCC}}$ to describe the dynamics on the timescale of C_t:

$$\begin{aligned}
\frac{d\widehat{C}}{d\hat{t}} &= \frac{Q_{C_t} V_{\text{S}}}{Q_C V_{\text{SOCC}}} (\bar{J}_{\text{diff}} + \bar{J}_{\text{leak}} - \bar{J}_{\text{SERCA}}) + \frac{Q_{C_t}}{Q_C} (\bar{J}_{\text{in}} - \bar{J}_{\text{pm}}), \\
\frac{d\widehat{C_t}}{d\hat{t}} &= \bar{J}_{\text{in}} - \bar{J}_{\text{pm}}, \\
\frac{d\widehat{C_b}}{d\hat{t}} &= \frac{Q_{C_t} V_{\text{S}} \gamma_1}{Q_C V_{\text{SOCC}}} (\bar{J}_{\text{IPR}} - \bar{J}_{\text{diff}}), \\
\frac{d\widehat{P}}{d\hat{t}} &= \frac{Q_{C_t} \psi_2}{Q_P V_{\text{SOCC}}} (\bar{V}_{\text{plc}} - \bar{V}_{\text{deg}}), \\
\frac{dm_{42}}{d\hat{t}} &= \frac{Q_{C_t} \lambda_{m_{42}}}{V_{\text{SOCC}}} (m_{42}^{\infty} - m_{42}), \\
\frac{dh_{42}}{d\hat{t}} &= \frac{Q_{C_t} \lambda_{h_{42}}}{V_{\text{SOCC}}} (h_{42}^{\infty} - h_{42}).
\end{aligned} \tag{2}$$

From numerical simulations of the model, we find that typical concentration scales for cytosolic Ca^{2+} and IP_3 are of the same order $Q_P = Q_C = 1$ μM. A typical concentration scale for total intracellular Ca^{2+} is of order $Q_{C_t} = 100$ μM. Substituting these scales and the values of parameters gives the following orders of magnitude of the right hand sides of system (2): $O(10^3)$, $O(1)$, and $O(10^5)$ for the $\widehat{C}$, $\widehat{C_t}$, and $\widehat{C_b}$ equations, respectively. The dynamics of $\widehat{P}$ has a timescale of order $O(10^2)$. m_{42} and h_{42} evolve on timescales of order $O(10^2)$ and $O(10)$, respectively. These indicate that the system contains multiple timescales, with $\widehat{C_b}$ evolving on the fastest and $\widehat{C_t}$ on the slowest.
